# Insights into the Genetic Variations of Human Cytochrome P450 2C9: Structural Analysis, Characterization and Comparison

**DOI:** 10.3390/ijms221910206

**Published:** 2021-09-22

**Authors:** Sonia J. Parikh, Sumit Kamat, Margaret Phillips, Samuel P. Boyson, Thomas Yarbrough, Dylan Davie, Qinghai Zhang, Karen C. Glass, Manish B. Shah

**Affiliations:** 1 Department of Pharmaceutical Sciences, Albany College of Pharmacy and Health Sciences, 106 New Scotland Avenue, Albany, NY 12208, USA; sonia.parikh@acphs.edu (S.J.P.); kamatsumit85@gmail.com (S.K.); Margaret.Phillips@uvm.edu (M.P.); samboyson@gmail.com (S.P.B.); Thomas.Yarbrough@acphs.edu (T.Y.); dylandavie2@gmail.com (D.D.); Karen.Glass@med.uvm.edu (K.C.G.); 2 Department of Pharmacology, Larner College of Medicine, University of Vermont, Burlington, VT 05405, USA; 3 Department of Integrative Structural and Computational Biology, The Scripps Research Institute, 10550 North Torrey Pines Road, La Jolla, CA 92037, USA; qinghai@scripps.edu

**Keywords:** cytochrome P450, drug metabolism, structural biology

## Abstract

Cytochromes P450 (CYP) are one of the major xenobiotic metabolizing enzymes with increasing importance in pharmacogenetics. The CYP2C9 enzyme is responsible for the metabolism of a wide range of clinical drugs. More than sixty genetic variations have been identified in CYP2C9 with many demonstrating reduced activity compared to the wild-type (WT) enzyme. The CYP2C9*8 allele is predominantly found in persons of African ancestry and results in altered clearance of several drug substrates of CYP2C9. The X-ray crystal structure of CYP2C9*8, which represents an amino acid variation from arginine to histidine at position 150 (R150H), was solved in complex with losartan. The overall conformation of the CYP2C9*8-losartan complex was similar to the previously solved complex with wild type (WT) protein, but it differs in the occupancy of losartan. One molecule of losartan was bound in the active site and another on the surface in an identical orientation to that observed in the WT complex. However, unlike the WT structure, the losartan in the access channel was not observed in the *8 complex. Furthermore, isothermal titration calorimetry studies illustrated weaker binding of losartan to *8 compared to WT. Interestingly, the CYP2C9*8 interaction with losartan was not as weak as the CYP2C9*3 variant, which showed up to three-fold weaker average dissociation constant compared to the WT. Taken together, the structural and solution characterization yields insights into the similarities and differences of losartan binding to CYP2C9 variants and provides a useful framework for probing the role of amino acid substitution and substrate dependent activity.

## 1. Introduction

Human cytochrome P450 (CYP) 2C9, a member of the CYP2C subfamily of CYP enzymes, is an important drug metabolizing enzyme and represents as much as ~20% of the total hepatic cytochrome P450 content [1,2,3]. It is predominantly responsible for the biotransformation of many prescribed clinical drugs that include anti-coagulant (S)-warfarin, anti-hypertensive losartan, anti-convulsant phenytoin, anti-diabetics tolbutamide and glimepiride, as well as the commonly used analgesic ibuprofen [4,5,6,7]. CYP2C9 is a highly polymorphic enzyme that includes over 60 classified genetic variants, with at least 20 reported to possess altered activity [8,9]. The two most characterized and commonly found variant alleles include CYP2C9*2 and CYP2C9*3, and both are associated with reduced enzyme function and altered drug metabolism [10,11]. However, the less characterized variants that include CYP2C9*5, *6, *8, and *11 are found with high frequency in populations of African origin, and they are more common than *2 and *3 alleles in these populations [12,13,14,15]. The CYP2C9*8 genetic variant, predominantly found in individuals of African ancestry with an allele frequency of ~0.06, is a missense variant (449G>A) that is mostly associated with reduced enzyme activity towards various drug substrates [16,17,18]. It represents an amino acid change from arginine to histidine at position 150, denoted as R150H. When individuals carrying two copies of this variant are exposed to high drug levels after standard doses, the mean oral clearance of (S)-warfarin exhibited significant reduction [18,19]. Reduced enzyme function was also observed with substrates such as phenytoin, however, data on the clinical and functional impact of the CYP2C9*8 allele are scant and suggest that this allele may exhibit substrate-dependent activity [12]. For example, in vitro and/or in vivo studies reported an increase in enzyme activity of CYP2C9*8 towards the sulfonylurea drug tolbutamide and no significant change in losartan oxidation in patients with this allele [14,15]. Such differences in substrate specificity may preclude the classification of this allele as a decreased function allele for sulfonylureas or drugs other than phenytoin or warfarin [20]. Although the role of CYP2C9*8 allele is less well characterized than the *2 and *3 alleles, it was considered as the gene of significant importance in pharmacogenomics due to its higher frequency in people of African ancestry, among whom warfarin is commonly used [20].

The amino acid arginine at 150 (R150) in CYP2C9 wild-type (WT) or histidine (R150H) in CYP2C9*8 is located on the surface of the protein at the D-helix region and away from the active site. The R150 in WT is highly conserved across different species (181 protein sequences) [21]. The effect of such distal amino acid change or R150H in CYP2C9*8 on the active site and differences in substrate binding and/or metabolism is not well understood. Interestingly, there are two additional variants, leucine and cysteine, that have been identified at position 150 with one being CYP2C9*27 representing R150L variation (Table 1) [22]. A recent study hypothesized the involvement of arginine or R150 in the salt bridge network with the neighboring residues, suggesting that the change to histidine at this solvent-exposed region may no longer coordinate similar ionic and electrostatic interactions and may result in destabilization of the structure [21]. The goal of the current study is to further our understanding of how genetic variations in CYP2C9, located away from the active site, affect drug binding and metabolism in the active site. We solved the structure of CYP2C9*8 by using X-ray crystallography in the presence of the drug substrate losartan. Losartan is a prodrug and an angiotensin II receptor antagonist that is metabolized by CYP2C9 and CYP3A4 (Supplementary Figure S1) [23,24]. In order to investigate the structure–function relationship of different genetic variations, we characterized the binding of losartan to CYP2C9 WT and CYP2C9*2 (R144C), *3 (I359L) and *8 variants using functional and solution biophysical methods that include thermal stability studies and isothermal titration calorimetry. The results demonstrated similarities and differences in the CYP2C9 structure and function upon binding to losartan and provide valuable knowledge that would help infer the effect of single nucleotide polymorphisms on the metabolism of a commonly prescribed medication.

## 2. Results and Discussion

### 2.1. Structure Determination and Comparison of CYP2C9*8 in Complex with Losartan

**Figure 1:**
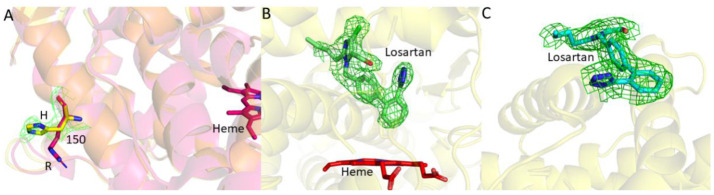
Electron density maps of CYP2C9*8 structure in complex with losartan (PDB ID 7RL2). (**A**) The electron density (*F_o_-F_c_* omit map in green mesh contoured at 3-*σ*) for histidine at position 150 is shown before adding the sidechain in the CYP2C9*8-losartan complex superimposed on the WT complex. (**B**,**C**) An unbiased *F_o_-F_c_* electron density omit maps contoured at 3-*σ* in green mesh after iterative refinement and before including losartan in CYP2C9*8 structure. Losartan is modelled in sticks in the observed electron density in (**B**) the active site and (**C**) the peripheral site. Heme is shown in red sticks.

**Figure 2:**
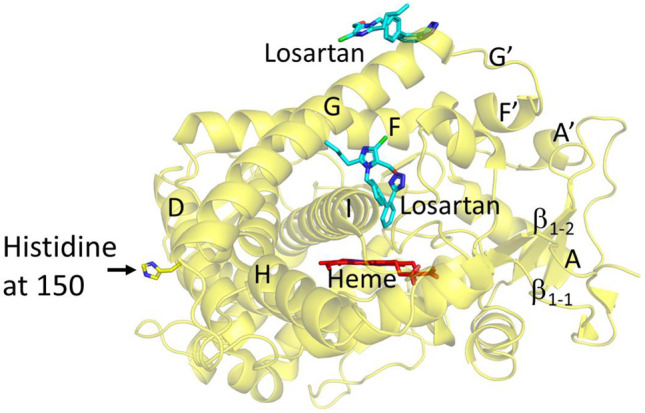
Structure of CYP2C9*8 genetic variant (yellow) in complex with losartan (PDB ID 7RL2). The losartan molecules, bound in the active site and the periphery, are shown as cyan sticks, and heme is shown by red sticks. The sidechain of the amino acid variation from arginine to histidine at position 150 or R150H is shown as stick representation.

**Figure 3:**
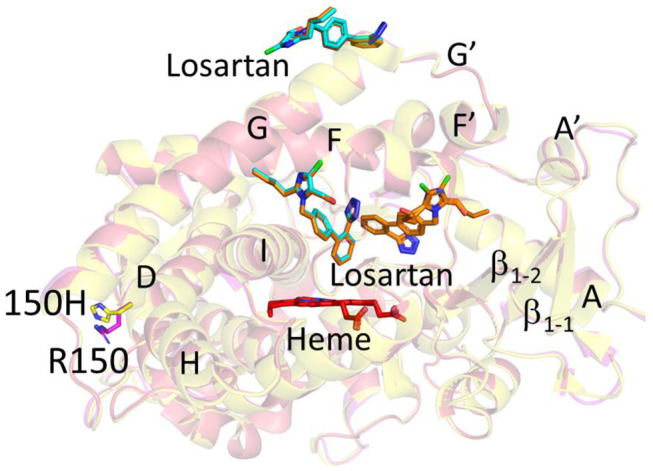
Overlay of CYP2C9*8 (yellow, PDB ID 7RL2) and CYP2C9 WT (magenta, PDB ID 5XXI) with losartan. The losartan molecules are shown as cyan sticks in *8 and brown sticks in WT structure. The third losartan observed only in WT is shown bound in the access channel. Heme is shown in red sticks. The sidechain of R150 in WT and 150H in *8 is represented by sticks.

The structure of CYP2C9*8 complexed with losartan clearly illustrates the amino acid change from R to H at position 150 (R150H) located on the surface of the protein (Figure 1A and Figure 2). The sidechain of histidine at 150 is oriented towards the solvent and away from other secondary structural elements. An unbiased electron density corresponding to losartan was observed in the active site and at the peripheral binding site (Figure 1B,C), and losartan was modeled into each of the electron densities prior to the final steps of refinement. Interestingly, the peripheral binding site of losartan observed in the *8 structure has been previously demonstrated in the published structures of CYP2C9 WT, *30 and *3 enzymes in complex with losartan (PDB IDs 5XXI, 5X23 and 5X24, respectively) [25]. The previously solved CYP2C9-WT (and *30) losartan complex illustrated the binding of three molecules of losartan, one in the active site, another at the peripheral site, and the third in the access channel. On the other hand, the CYP2C9*8-losartan complex showed two molecules of losartan that include binding at the active site and the peripheral site, as observed in the WT complex, but lost one binding site in the access channel [25]. The orientation of losartan in the active site and at the peripheral binding site is similar to that observed in the WT complex. As shown in Figure 3, the structural overlay of CYP2C9*8 with the WT complex of losartan revealed the similarities and differences between the two structures. The overall fold was conserved between the two structures with a root mean square deviation (RMSD) of 0.23 Å; however, the structures differed in the occupancy of losartan molecules. Furthermore, the structure of the *8 complex demonstrated the rotation of the H150 sidechain by about ~60° in an alternate conformation compared to the sidechain of R150 in the WT complex. On the other hand, other amino acid residues in the region surrounding the R150H variation overlaid well between both structures. The active site losartan has a biphenyl ring located near the heme, while the tetrazole and the imidazole rings were oriented near the helices away from the heme. A water molecule was found ordered near the heme iron, as observed in the WT complex.

**Figure 4:**
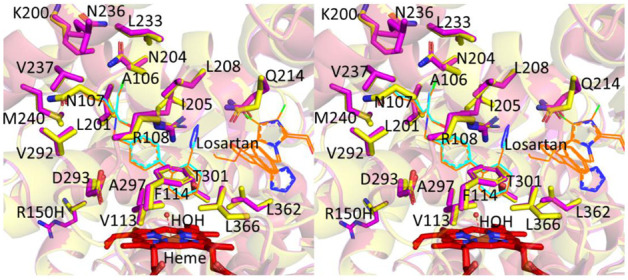
Active site overlay of CYP2C9*8 (yellow, PDB ID 7RL2) and CYP2C9 WT (magenta, PDB ID 5XXI) in stereo view showing residue sidechains in sticks located within 5 Å of losartan. The losartan molecules are shown as thin lines in cyan (*8) and brown (WT). Heme is shown as red sticks. The sidechains of R150 in WT and 150H in *8 located far away from the active site are shown as sticks.

The structural overlay of the active site of the *8 and the WT complex of losartan is shown in the Figure 4, which illustrates the amino acid residue sidechains within 5 Å of losartan. The residue sidechains of R108 and N204, previously shown to be important in substrate hydroxylation, interact with losartan in the active site. As observed in the WT complex, the losartan bound on the surface or peripheral binding site interacts with the amino acid residue sidechains at positions 226 to 229 that include phenylalanine, proline, glycine and threonine or FPGT located within 5 Å of losartan [25]. The chlorine group of losartan interacts with the lysine at position 232. The conserved FPGT amino acid sequence from position 226 to 229 is observed across other human CYP2C subfamily of enzymes that include CYP2C8, 2C9 and 2C19. Such peripheral site in CYP2C8 illustrated the binding of a palmitic acid molecule at this region that interacted with the same residues on the surface [26]. The binding of ligands at this region, as observed in multiple structures that include CYP3A4, may suggest the potential role of this site in substrate recognition [27]. Overall, while the structures of CYP2C9 WT and *8 complexed with losartan are highly conserved, there was a notable difference in the number of losartan molecules bound, which could explain differences in activity towards the drug.

**Figure 5:**
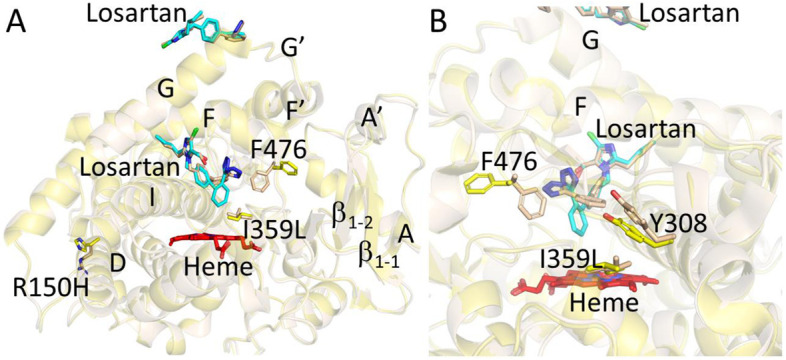
Structural overlay of CYP2C9*8 (yellow, PDB ID 7RL2) and CYP2C9*3 variant (wheat, PDB ID 5X24) in complex with losartan. Losartan molecules are shown as cyan sticks in *8 and wheat sticks in *3 complex. The R150H variation in *8 and the I359L variation in *3 are shown as stick representations. The sidechain of F476 is shown in sticks in both structures. Heme is show in red. (**A**) Overall view of the superimposed structures. (**B**) An alternate view of the structural overlay focused near the I359L site. The sidechain of residue Y308 is shown in sticks.

**Figure 6:**
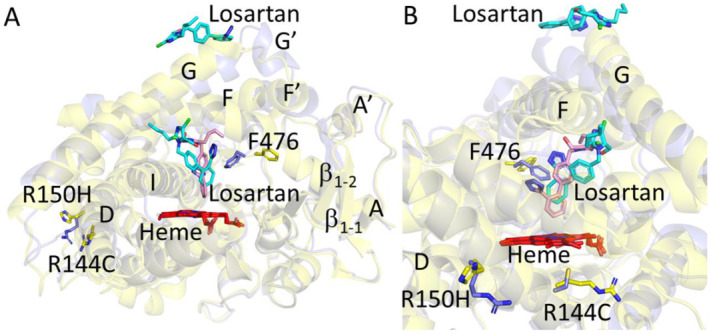
Structural overlay of CYP2C9*8 (yellow, PDB ID 7RL2) and CYP2C9*2 variant (blue, PDB ID 6VLT) in complex with losartan. Losartan molecules are shown as cyan and pink sticks in *8 and *2 complex, respectively. The R150H variation in *8 and R144C variation in *2 is shown as stick representation. The sidechain of F476 is shown in sticks in both the structures. Heme is show in red. (**A**) Overall view of the superimposed structures. (**B**) An alternate view of the structural overlay focused near the site of amino acid variations, R144C and R150H.

**Figure 7:**
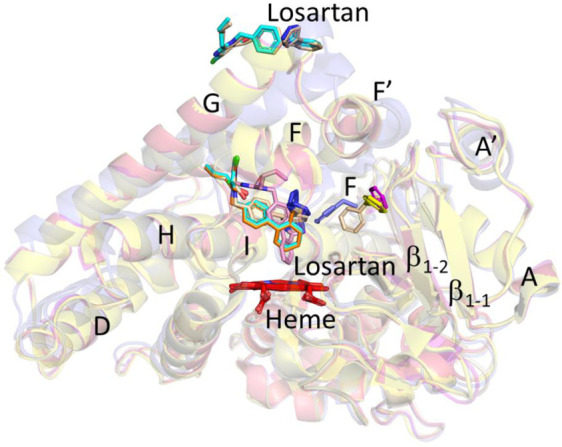
The structural overlay of CYP2C9*8 (yellow, PDB ID 7RL2), *2 (blue, PDB ID 6VLT), *3 (wheat, PDB ID 5X24) and WT (magenta, PDB ID 5XXI) complexes with losartan. The differences in the conformation of F476 sidechain is shown in each of the CYP2C9 structures. The orientation of the losartan molecule is shown in cyan (*8), pink (*2), wheat (*3) and orange (WT) in the corresponding structures. The additional losartan molecule observed in the WT structure in the access channel is not shown for the sake of clarity.

Structural analysis of the CYP2C9*3-losartan complex had shown two molecules of losartan with one bound on the surface in a similar orientation as observed in the WT and the current *8 complex [25]. However, the binding of losartan in the active site differed with the biphenyl ring rotating ~90° in the *3 complex compared to the losartan in the active site of *8 or other losartan complexes. Both *3 and *8 were superimposable with an RMSD of 0.29 Å (Figure 5A). An important difference included the effect of amino acid substitution in *3 (isoleucine to leucine at 359 or I359L) that perturbs the sidechain conformation of tyrosine at 307, as discussed previously [25]. Such movement of Y307 sidechain seems to affect the *β_4_* loop, where the sidechain of F476 in the *3 complex rotates by ~90° into the access channel (Figure 5B). It is in this access channel region that the third molecule of losartan is bound in the WT complex but not in the *3 or the *8 structures. In addition, the structural overlay of the CYP2C9*8 with the recently solved CYP2C9*2 (R144C)-losartan complex revealed changes in the overall conformation (RMSD ~0.71 Å) that was largely reflected in the F-G cassette, which comprises the F, F’, G’ and G helices (Figure 6A) [28]. The differences in the conformation between the *8 and the *2 complexes were similar to those observed between the WT and the *2 complexes of losartan; however, the losartan in the access channel was not observed in both (*8 and *2) structures. Furthermore, the losartan binding site at the periphery was unclear in the *2 complex but not in the other losartan complexes solved thus far [25,28]. Figure 6B shows the distinct orientation of losartan in the active site and differences in the secondary structural elements at position R144C between the two structures that were consistent with the WT complex. The sidechain of F476 in the *2 complex protrudes into the access channel in a similar manner, as observed with the *3-losartan structure. Interestingly, the structural overlay of CYP2C9*2, *3, *8 and WT complexes of losartan (Figure 7) revealed that the F476 sidechain in the *8 structure rotates ~30° towards the access channel compared to that observed in the WT structure. The sidechain of F476 in *8 is observed in the conformation that is in between the F476 conformations of the *3 or *2 and WT complexes with losartan. Taken together, the rotation of the F476 sidechain towards the access channel region in these structures yields insights into the effect on the binding of losartan in this region.

Previously solved losartan complexes of the CYP2C9 WT and the *30 variant were superimposable in a structural overlay [25]. Thus, the differences noted between the *8 and the *30 complexes of losartan were consistent with those observed with the *8 and the WT complex (Supplementary Figure S2A). The *30 variant represents the amino acid substitution from alanine to threonine at position 477 (A477T). As described previously, the amino acid sidechain of glutamine at 214, or Q214, near the access channel reorients to a hydrogen bond with the polar substitution, or T477, in the *30 variant [25]. On the other hand, the Q214 sidechain in the WT complex interacts with the third losartan in the access channel. The conformation of the Q214 sidechain in the *8 complex, which lacks such additional losartan in the access channel, is similar to the WT complex. Losartan in the active site and at the peripheral site was bound in the same fashion in both *8 and *30 structures. Furthermore, the *8-losartan complex was compared to the (S)-warfarin bound structure of CYP2C9 (RMSD 0.29 Å), which was the first published structure of this drug metabolizing enzyme (Supplementary Figure S2B). A single (S)-warfarin molecule was bound in the access channel region but not in the active site or the peripheral site. The WT-losartan and the (S)-warfarin complexes of CYP2C9 had drug bound in this access channel, and the sidechain conformation of F476 was altered or pushed away from this region. On the other hand, the *2, *3 or *8, with no ligand at this site, had the sidechain protruding the access channel to a different extent, as discussed earlier. Furthermore, there were some differences in the helices near the periphery that may be attributed to the engineered amino acids in the WT construct used to crystallize the (S)-warfarin complex of CYP2C9. Overall, even though the conformation of CYP2C9*8-losartan complex was indistinguishable from several of the previously solved structures of CYP2C9-losartan complexes, there were noteworthy differences in losartan binding and orientation of important amino acid sidechains.

### 2.2. Functional Characterization of Losartan Binding to CYP2C9 WT and Variants

**Figure 8:**
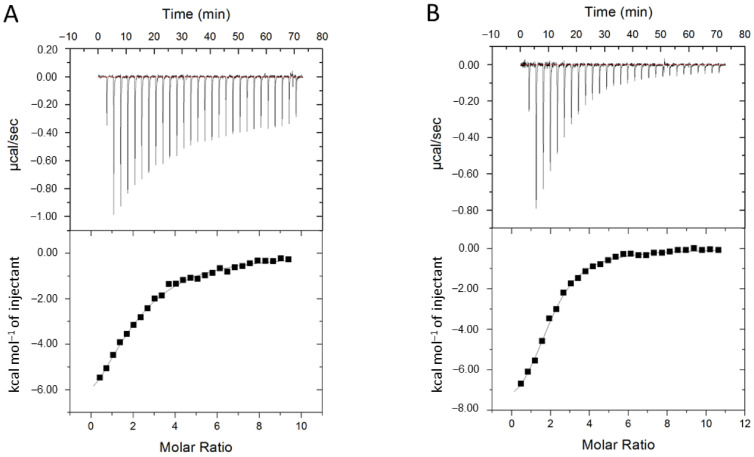
Isothermal titration calorimetry (ITC) binding isotherms for the CYP2C9*3 and CYP2C9*8 variants with losartan. The exothermic enthalpy plots were used to calculate the average apparent dissociation constant (K_D_) for losartan binding to (**A**) CYP2C9*3 and (**B**) CYP2C9*8. Standard deviation was calculated from the average of three independent experiments.

The binding of losartan in solution with CYP2C9*3 and *8 variants was elucidated by using ITC and compared with the previously published titrations of losartan with CYP2C9 WT and the *2 variant (Figure 8). The CYP2C9*3 variant illustrated significant reduction in binding affinity to losartan compared with WT and to the *2 variant. The average apparent dissociation constant (*K_D_*) value for losartan binding to CYP2C9*3 was 24.5 ± 2.7 µM, which was more than three-fold weaker than the WT enzyme with a previously published *K_D_* value of 6.8 ± 0.7 µM for losartan binding [28]. On the other hand, the average *K_D_* for losartan binding to CYP2C9*8 was 8.95 ± 0.97 µM, which was comparable to that yielded previously by the *2 variant (9.0 ± 0.5 µM). Differential Scanning Fluorimetry (DSF) was performed in order to measure the thermal denaturation or “melting” point (*T_m_*) of CYP2C9 WT and the *2, *3 and *8 variants using SYPRO Orange in the presence and absence of losartan in similar buffer conditions as with ITC. The results show that there are small differences between the thermal stability of the ligand-free WT and variant proteins (Supplementary Figure S3 and Table S1). For example, the *2 and *3 variants have a stabilizing effect on the CYP2C9 structure as indicated by a slight increase in *T_m_* of the ligand-free proteins, while the *T_m_* of the *8 variant is not significantly different from the ligand-free WT protein. These results indicate that the overall fold of the protein is preserved, and they are consistent with our crystal structure, which indicated no significant differences in the overall fold or conformation of the *8 variant compared to WT. Thus, the observed differences in metabolism by these CYP2C9 variants do not appear to be the result of a structural destabilization due to loss of ionic and electrostatic interactions as previously speculated [21]. In addition, we only observed small changes to the *T_m_* of WT and variant proteins in the presence and absence of losartan. Similarly, this result indicates that the binding of losartan molecules does not cause any notable change in the stability of the CYP2C9 enzymes.

Furthermore, a reconstituted system containing the redox partner cytochrome P450 reductase and losartan substrate was used to determine the activity of CYP2C9 variants by the consumption of NADPH. The rate of NADPH consumption was determined by monitoring the change in absorbance at 340 nm as a function of time. The recombinant CYP2C9 WT incubations resulted in an NADPH depletion rate that was markedly higher than the same noted for the CYP2C9*3, which displayed an even lower consumption of NADPH or losartan turnover than the *2 variant noted previously (Supplementary Figure S4). The CYP2C9*8 variant showed decreased ability to turn over losartan compared to the WT enzyme; however, its activity was not as significantly reduced as it was for the *3 variant.

In conclusion, the crystal structure of CYP2C9*8 was solved in the presence of an angiotensin II receptor blocker drug losartan. Losartan was bound in the active site and on the periphery or surface in an orientation that is consistent to that observed in the previously solved WT-complex with losartan. The new structure differed with only two molecules of drug bound and no ligand was observed in the access channel, as was observed with the WT complex. The enzymatic characterization of different CYP2C9 variants to determine the binding affinity, stability and turnover of losartan in comparison with the WT enzyme yielded insights into the effect of amino acid changes that occur at various locations on drug metabolism. As noted earlier, a recent hypothesis suggested the involvement of R150 in coordinating ionic and electrostatic interactions, and the change to histidine in the *8 variant may affect such interactions [21]. Another hypothesis includes the change in protein–protein interaction with the redox partner cytochrome P450 reductase as the location at 150 is in close vicinity of the site previously illustrated to be involved in binding with the redox partner P450 reductase [29]. Whether such loss or change of interactions due to R150H substitution in the *8 affected the binding of third losartan in the access channel remains to be elucidated. One of the differences includes the change in conformation of the residue sidechain F476 in the current structure. Even though the rotation of this sidechain was not as profound as that observed in the *3 or *2 complexes, the crystallographic results suggest the importance of the role of the F476 sidechain in ligand binding. Moreover, the effect of crystallization buffers and conditions in any given crystallography work cannot be ignored. Importantly, the orientation of losartan in the active site of CYP2C9*8 is inconsistent with the demonstrated metabolism as the site of hydroxylation of losartan is located further away from the heme iron. Such an altered pose has been observed with several crystal structures of P450 complexes, including the losartan complexes of CYP2C9 described previously [25,28]. The crystallization is performed in the absence of NADPH or cytochrome P450 reductase, and the substrate may likely reorient to a catalytically favorable pose in the presence of the redox partner during the catalytic cycle. More studies are needed to probe the effect of these redox partners on the orientation of the ligand that is conducive to metabolism.

In the clinical context, the current pharmacogenomics annotations indicates that the patients carrying the CYP2C9*8 allele in combination with another decreased functional allele may demonstrate reduced metabolism of losartan compared to the patients possessing two WT or normal function alleles [30]. However, clear evidence regarding the impact on the metabolism of losartan and the role of *8 allele (*8/*8) and in combination with a WT allele (*1/*8) compared to patients with two normal function alleles is scant, with the exception of a study in 2004 [14]. Furthermore, two promoter variants in strong linkage disequilibrium with CYP2C9*8 have been linked to decreased CYP2C9 expression and may indicate the effect of this allele [17]. As noted previously, clinicians should be aware of the concern of substrate-dependent activity when interpreting genetic results that include CYP2C9*8 and use caution regarding any clinical recommendation for substrates other than warfarin or those that have been extensively characterized [20]. Thus, further structural, functional and pharmacogenomic studies in the presence of different substrates are warranted in order to elucidate substrate specificity and the influence of the distal variation on the surface.

## 3. Materials and Methods

### 3.1. Materials

Tryptone enzymatic digest, yeast extract, glycerol, potassium phosphate dibasic, potassium phosphate monobasic, L-histidine monohydrochloride, ampicillin sodium salt, phenylmethylsulfonyl fluoride (PMSF), sodium chloride, ethylenediaminetetraacetic acid (EDTA), chloramphenicol, Terrific Broth, deoxyribonuclease I and ribonuclease A were purchased from Sigma-Aldrich (St. Louis, MO, USA). Difco Luria-Bertani broth lennox from Becton Dickinson (Franklin Lakes, NJ, USA). Isopropyl β-F-1-thiogalactopyranoside (IPTG) Dioxane-Free and 5-aminolevulinic acid hydrochloride were obtained from BOC Sciences (Upton, NY, USA). CHAPS(2-2-Cholamidopropyldimethylammonio)-1-propanesulfonate and Tris(2-carboxyethyl)phosphine hydrochlorate (TCEP) were obtained from Soltec venture (Beverly, MA, USA). HisPur Ni-NTA resin was from ThermoFisher Scientific (Waltham, MA, USA). CM Macroprep was from Bio-Rad (Hercules, CA, USA). 5-Cyclohexyl-1-hexylpentyl-β-D-maltoside (CYMAL-5) was obtained from Anatrace (Maumee, OH, USA). The 3*α*-Hydroxyl-7*α*,12*α*-di-(((2-(trimethylamino)ethyl)phosphoryl)ethyloxy)-cholane (FA-7/234-chol) is a facial amphiphile used as described previously [31]. PEGRx screens were obtained from Hampton Research. Human cytochrome P450 reductase was obtained from OriGene (Rockville, MD, USA).

### 3.2. Protein Expression and Purification

The CYP2C9*8 (R150K), CYP2C9*2 (R144C) and CYP2C9*3 (I359L) were generated from the CYP2C9 wild-type construct, which was a gift from Dr. Keiko Maekawa, Doshisha Women’s College of Liberal Arts, Kyoto, Japan. As described previously, the amino acid residues from 1 to 23 in the CYP2C9 sequence that represents the N-terminal transmembrane anchor domain were replaced with residues MAKKT, and the C-terminal or last residue at position 490 was mutated from valine to isoleucine (V to I), followed by a His-tag or four histidine residues for purification purposes [25]. The altered cDNA was inserted into the pTrc expression vector. Site-directed mutagenesis was performed in order to generate appropriate CYP2C9 variants using the QuikChange II Site Directed Mutagenesis Kit (Agilent), and the mutation was confirmed by DNA sequencing. The resulting plasmid was transformed into *E. coli* Rosetta2 competent cells, which supply tRNAs for rare codons on a compatible chloramphenicol-resistant plasmid. Autoclaved Luria-Bertani broth, supplemented with chloramphenicol (25 μg/mL) and ampicillin (100 μg/mL), was inoculated with a plasmid glycerol stock of CYP2C9*8, *2 or *3 and grown overnight in a shaking incubator at 225 rotations per minute (RPM) and 37 °C. The following day, autoclaved Terrific Broth, supplemented with chloramphenicol (25 μg/mL) and ampicillin (100 μg/mL) as well as TB salts, was inoculated with 15 mL of the overnight culture. The culture was subsequently grown at 37 °C with shaking at 230 RPM until reaching an optical density at 600 nm wavelength of 0.7–0.8. The protein expression was induced by adding IPTG and 5-aminolevulinic acid at 1 mM and 0.5 mM, respectively. The cultures were incubated in the shaking incubator at 30 °C and 190 RPM for 65 h. *E. coli* cells were harvested by centrifugation at 4000× *g* for 10 min at 4 °C.

The pellet was resuspended in 10% original culture volume (400 mL for 4 L culture) with 20 mM potassium phosphate (pH 7.4), 20% (*v*/*v*) glycerol, 1.25 mM TCEP and 1 mM PMSF. Lysozyme (0.3 mg/mL) was added to the resuspended cells and stirred for 30 min at 4 °C, followed by centrifugation at 5640× *g* for 30 min. The spheroplasts were resuspended in 5% original culture volume in 500 mM potassium phosphate, 20% (*v*/*v*) glycerol, 1.25 mM TCEP, 0.5 mM PMSF, ribonuclease A (10 μg/mL) and deoxyribonucelase (10 μg/mL). The homogenized mixture was sonicated at 40 pulses per minute for 1 min for a total of four minutes. The detergent CHAPS was added (0.8% final concentration), and the lysate was stirred for 90 min (4 °C) followed by centrifugation at 33,000× *g* for 2 h at 4 °C.

Equilibration of the nickel NTA resin was performed by using 50 mM potassium phosphate buffer with 20% (*v*/*v*) glycerol (4 °C, pH 7.4). The protein supernatant was added to the equilibrated resin and stirred overnight to promote protein binding. The protein-bound resin was collected by centrifugation for 3 min at 1940× *g* and added to a Bio-Rad Econo-Column. The Bio-Rad NGC Quest chromatography system was used to wash the resin on the column with ten column volumes of 100 mM potassium phosphate buffer with 20% glycerol, 0.5 mM PMSF, 2 mM TCEP, 100 mM NaCl, 0.5% CHAPS and 5 mM histidine. The protein was eluted with 10 mM potassium phosphate, 20% glycerol, 100 mM NaCl, 2 mM TCEP, 0.5 mM PMSF, 0.5% CHAPS and 50 mM histidine at a rate of 1.5 mL/min. Using a UV spectrophotometer, the absorbance of each of the collected fractions was measured at 415 nm and 280 nm. Those fractions with a 415/280 ratio greater than 1 were pooled, and the concentration was measured. The pooled protein was diluted 5 times (by volume) with a 5 mM potassium phosphate buffer with 20% glycerol, 0.5 mM PMSF, 2 mM TCEP, 1 mM EDTA and 0.5% CHAPS.

Equilibration of the weak cation exchange resin, CM Macroprep (Bio-Rad), was performed using 10 mM potassium phosphate buffer with 20% glycerol (1 mL resin/100 nmol protein). The CM resin was incubated overnight with the diluted protein (4 °C). The resin was collected by centrifugation at 2880× *g* and added to a Bio-Rad econo-column and washed with ten column volumes of buffer containing 5 mM potassium phosphate, 20% glycerol, 1 mM EDTA, 2 mM TCEP and 50 mM NaCl. The protein was eluted using a buffer containing 50 mM potassium phosphate, 20% glycerol, 2 mM TCEP, 1 mM EDTA and 500 mM NaCl at a flow rate of 1 mL/min and collected in 1 mL fractions. The absorbances at 415 nm and 280 nm were measured, and the fractions with 415/280 nm of 2 or greater were pooled in a falcon tube before measuring the concentration. The reduced CO difference analysis using dithionite illustrated the peak at 450 nm in the presence of carbon monoxide and absent at 415–420 nm.

### 3.3. Crystallization

The pooled protein was diluted to 18 μM using the sucrose buffer (50 mM potassium phosphate, 500 mM NaCl, 0.5 M sucrose, 1 mM EDTA and 0.2 M PSMF). A stock solution of losartan, dissolved in water, was prepared at 40 mM concentration and added to a final concentration of 180 μM. The protein–ligand solution was incubated on ice overnight. The following day, the protein–ligand complex was concentrated 10 times by volume using Amicon Ultra centrifugal filters (10 kDa molecular weight cut off) and then diluted once again to 18 μM with the addition of losartan to 180 μM. This concentration/dilution process was repeated twice more. The protein–ligand complex was then concentrated to a final concentration of 500 μM. CYMAL-5 (detergent) was added to 4.8 mM final concentration followed by the addition of 234-chol facial amphiphile (0.077% *w*/*v*). Finally, losartan was added to a final concentration of 1 mM and the complex was filtered prior to crystallization using the Amicon filtration units. Crystal screening was performed using sitting drop vapor diffusion where drops were equilibrated against 50 μL well-reservoir by mixing screen solution (PEGRx from Hampton Research) and CYP2C9*8-losartan complex in a 1:1 ratio. The plates were incubated at 18 °C, and the crystals of CYP2C9*8 grew over the course of 1–2 weeks in drops containing 0.1 M HEPES pH 7.5 and 30% *w/v* Polyethylene glycol 1000. Crystals for the CYP2C9*8-losartan complex were collected in a crystallization buffer by using 20% sucrose as cryo-protectant, and then it was flash frozen in liquid nitrogen before shipping them to the Stanford Synchrotron Radiation Light source.

### 3.4. Structure Determination and Refinement

The crystals diffracted to 2.2 Å resolution, and the crystallographic data were collected remotely by using beam line 12-2 [32,33,34]. Data were collected using 1-degree oscillations (360 frames) and 5 s exposure at 100 K, and the images were integrated using iMosflm and scaled using SCALA [35,36,37,38]. The automated molecular replacement program Balbes was used with Protein Data Bank ID 1OG5 as the search template [39]. The space group was determined, and one molecule in the asymmetric unit was found. The crystallographic software COOT was used to build the model using the CYP2C9 protein sequence, and the *2F_o_-F_c_* and *F_o_-F_c_* electron density maps were contoured to 1-σ and 3-σ, respectively [40]. The REFMAC5 program was used for refinement, and the R-factor of 0.17 and R-free of 0.22 were achieved [41]. A total of 125 water molecules and a potassium and a phosphate molecule were present in the structure. Crystallographic data collection and refinement statistics are shown in Supplementary Table S2. The following amino acid sidechains demonstrated disordered electron density: L43, K48, K138, K185, Q193, E199, K235, K275, H276, N277, Q278, E285, E328, R333, N334, N378, M406, K421, L461, K465, N466 and I490. The coordinates and structure factors with no Ramachandran outliers and clash score and MolProbity score of 92 and 88 percentile, respectively, were submitted to the Protein Data Bank (PDB 7RL2) [42]. All figures of protein structures were made using PyMOL, the PyMOL Molecular Graphics System Version 2.0, Schrodinger, LLC. (Portland, OR, USA).

### 3.5. NADPH Consumption Assay

Enzymatic assays were performed using 50 pmol reconstituted CYP2C9 (WT, *3 and *8) incubated with 50 pmol human cytochrome P450 reductase on ice for 5 min [28,43]. An amount of 10 µM losartan was added, and the reaction was further incubated on ice for 5 min. An amount of 50 mM potassium phosphate buffer (pH 7.4) was added to a final volume of 95 µL and incubated at 37 °C for 5 min. NADPH (0.5 mM) was added to initiate the reaction and was immediately diluted to 1 mL (in phosphate buffer). The absorbance was measured every 0.25 min for 5 min using ThermoScientific Biomate 160 UV-vis spectrophotometer. The rate of NADPH was calculated by using an extinction coefficient of 6.22 mM^−1^cm^−1^ and expressed as nmol NADPH consumed per minute per nanomole of CYP2C9. Control experiments were prepared without losartan or P450 reductase, and a blank was prepared without NADPH. The assays were repeated in triplicates, and standard deviation was expressed as error bars.

### 3.6. Thermal Stability Assays

Differential Scanning Fluorimetry was performed in a 96-well clear plastic plate using an Eppendorf Master cycler Realplex2 qPCR machine to monitor thermally induced protein denaturation by measuring changes in fluorescence of a dye SYPRO Orange, which binds to hydrophobic regions of proteins exposed by unfolding. The CYP2C9 WT, *2, *3 and *8 protein samples were in the buffer containing the 50 mM potassium phosphate, pH 7.4, 500 mM NaCl, 1 mM EDTA and 1 mM TCEP. Two control experiments included are (1) the buffer with 5 μM SYPRO orange dye and (2) the buffer with 5 μM SYPRO orange dye with 50 μM losartan. The experimental setup included samples with or without losartan. One set of samples included the WT or *2 or *3 or *8 with 5 μM SYPRO orange dye in the buffer, and another set of samples included the WT or *2 or *3 or *8 with 5 μM SYPRO orange dye and 50 μM losartan in the buffer. Each sample was made to a total volume of 300 μL. Each well had 98 μL sample volume, and the assay was performed in triplicates. The plate was sealed with an optically clear adhesive tape to prevent sample loss during heating. The temperature gradually ramped up from 25 to 95 °C while monitoring the change in fluorescence intensity of the dye. GraphPad Prism software was used to analyze the data and used to calculate the melting point of the protein sample by applying nonlinear regression using the melting Boltzmann equation. The standard error was calculated from triplicates of two individual experiments.

### 3.7. Isothermal Titration Calorimetry

Isothermal titration calorimetry (ITC) data were obtained using a MicroCal ITC200 (Malvern Panalytical, Westborough, MA, USA). Experiments were performed at 25 °C with 900 μM losartan in the syringe titrated into 83 μM CYP2C9*8 or *3 in the sample cell. CYP2C9*8 or *3 was snap-frozen in liquid nitrogen for storage after purification and then thawed out and dialyzed into ITC buffer containing 50 mM potassium phosphate, 500 mM NaCl, 1 mM EDTA and 1 mM TCEP at pH 7.4. Losartan (soluble in water) was diluted to 900 μM for titration using the ITC dialysis buffer. ITC experiments were performed by titrating a total volume of 40 μL of losartan in the syringe into 200 μL of CYP2C9*8 or *3 in the sample cell over the course of 28 injections. The first injection was 0.5 μL followed by a delay of 180 s. Subsequently, 27 injections of 1.4 μL with a delay of 150 sec between injections were titrated into the sample cell. All injections had a duration time of 1 s. The syringe paddle was constantly spinning at 750 RPM in the sample cell for the duration of the experiment. ITC binding isotherms were analyzed using Origin 7.0552 software (OriginLab Corporation, Northampton, MA, USA) in order to perform a Marquandt nonlinear least squares analysis assuming one set of sites model. The initial injection was omitted from the *K_D_* and N values. The active fraction of CYP2C9*8 or *3 in the sample cell was calculated based on a 2:1 ratio for the enzyme in complex with losartan as observed in the crystal structure of these variants. The raw data were integrated, baseline adjusted, normalized for active fraction concentration, and then analyzed with a one set of sites binding model. The experiments were performed in triplicate, and the overall method was consistent with the experiments performed previously with CYP2B6 [28,44].

## Figures and Tables

**Table 1:** CYP2C9 WT and variants discussed in the current manuscript are shown with their respective amino acid changes.

Cytochrome P450	Amino Acid Change
CYP2C9 WT (wild-type)	None
CYP2C9*2	R144C
CYP2C9*3	I359L
CYP2C9*5	D360E
CYP2C9*6	Frameshift
CYP2C9*8	R150H
CYP2C9*11	R335W
CYP2C9*27	R150L
CYP2C9*30	A477T

## Data Availability

The coordinates and structure factors for the CYP2C9*8-losartan complex were submitted to the Protein Data Bank (PDB ID 7RL2).

## Supplementary Materials

The following are available online at https://www.mdpi.com/article/10.3390/ijms221910206/s1, Figure S1: Metabolism scheme of the prodrug losartan to the active form E3174 primarily catalyzed by CYP2C9. Figure S2: Structural overlay of CYP2C9*8-losartan complex. Figure S3: Thermal stability assay of CYP2C9 WT and the variants. Table S1: Melting temperatures of CYP2C9 WT, *2, *3 and *8 in the absence (apo) or presence of the drug substrate losartan in a 1:10 molar ratio. Figure S4: The amount of NADPH consumed in nmol per minute per nmol CYP2C9 enzymes in the presence of 10 µM losartan. Table S2: Crystallographic data collection and refinement statistics.

## Abbreviations

CYP	Cytochrome P450
ALA	5-aminolevulinic acid hydrochloride
NADPH	Nicotinamide adenine dinucleotide phosphate
PDB	Protein Data Bank
EDTA	Ethylenediaminetetraacetic acid
IPTG	Isopropyl β-F-1-thiogalactopyranoside
CYMAL-5	5-Cymal-hexylpentyl-β-D-maltoside
CHAPS	(2-2-Cholamidopropyldimethylammonio)-1-propanesulfonate
TCEP	Tris (2-carboxyethyl) phosphine hydrochloride
RMSD	Root mean square deviation
PMSF	Phenylmethylsulfonyl fluoride
ITC	Isothermal titration calorimetry.
