# Supplementary material for: Insights into the Genetic Variations of Human Cytochrome P450 2C9: Structural Analysis, Characterization and Comparison
Source: Int J Mol Sci. 2021 Sep 22;22(19):10206. doi: 10.3390/ijms221910206 (PMC8508694; doi:10.3390/ijms221910206)
Supplement: Supplementary file 1 [file ijms-22-10206-s001.zip › ijms-1374019-supplementary.pdf]

Insights into the Genetic Variations of Human Cytochrome P450 2C9: Structural Analysis, Characterization and Comparison.

Sonia J. Parikh [1], Sumit Kamat [1,#], Margaret Phillips[1,3,#], Samuel P. Boyson [1], Thomas Yarbrough [1], Dylan Davie [1], Qinghai Zhang [2], Karen C. Glass [1,3] and Manish B. Shah [1,*]

[1]Department of Pharmaceutical Sciences, Albany College of Pharmacy and Health Sciences, 106 New Scotland Avenue, Albany, NY 12208, USA. [2]Department of Integrative Structural and Computational Biology, The Scripps Research Institute, 10550 North Torrey Pines Road, La Jolla, CA 92037 USA. [3]Department of Pharmacology, Larner College of Medicine, University of Vermont, Burlington, VT 05405, USA. [#]Contributed equally.

Corresponding author: Manish B. Shah, Department of Pharmaceutical Sciences, Albany College of Pharmacy and Health Sciences, 106 New Scotland Avenue, Albany, NY 12208, USA. E-mail: manish.shah@acphs.edu, Phone: 518 694 7343, Fax: 518 694 7499.

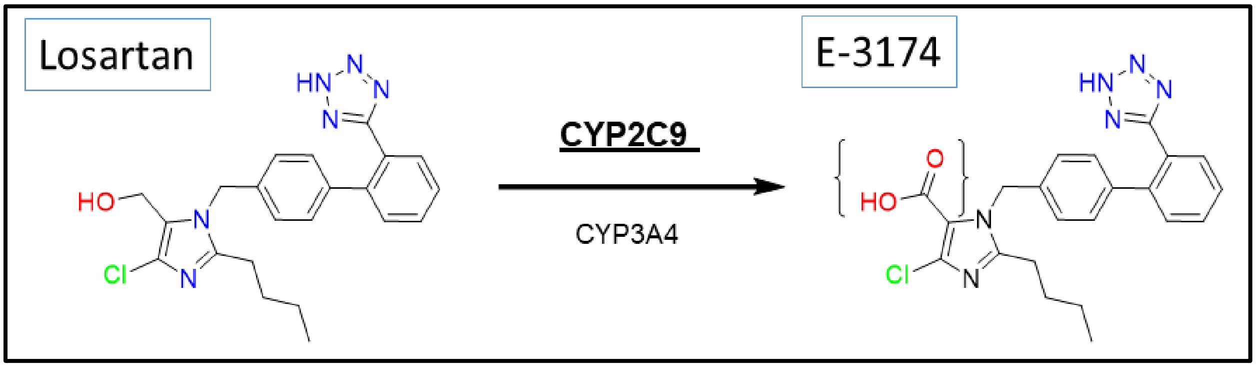

Figure S1: Metabolism scheme of the prodrug losartan to the active form E3174 primarily catalyzed by CYP2C9**.** Structures were made using ChemDraw Professional 15.1.

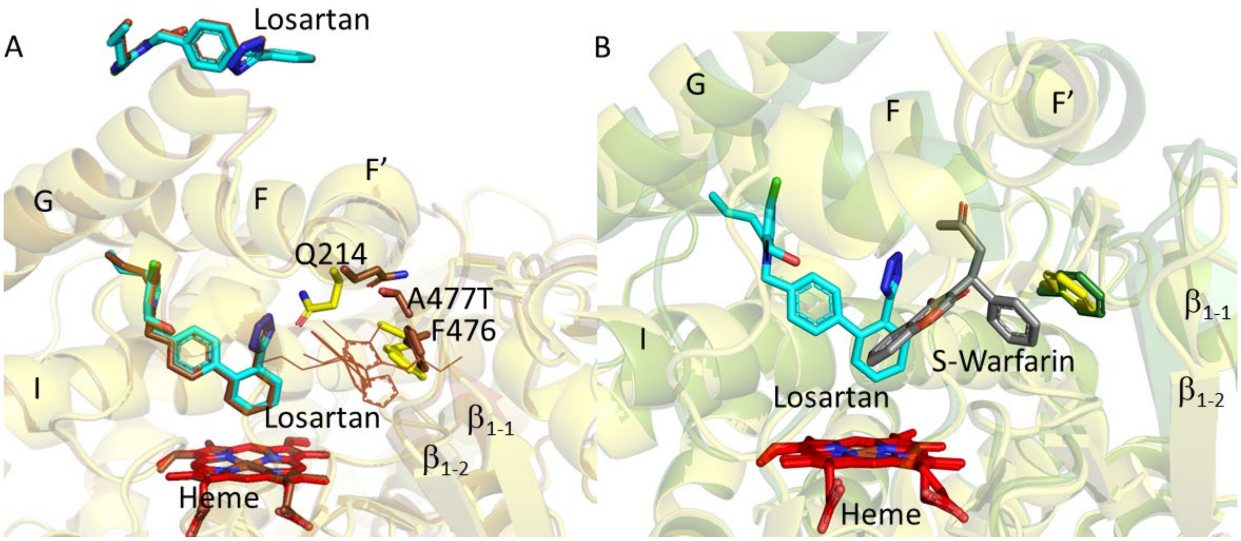

Figure S2: Structural overlay of CYP2C9*8-losartan complex. (A) Structure of the *8 variant (yellow, PDB ID 7RL2) superimposed on to the CYP2C9*30 complexed with losartan (brown, PDB ID 5X23) of CYP2C9. The losartan in *8 structure is shown in cyan sticks, whereas in the *30 structure is shown in brown sticks. The losartan in the access channel of the *30 structure is shown in thin lines for clarity. The sidechains of Q214 and F476, and amino acid substitution at A477T (*30) is shown in sticks. (B) Structure of the *8 variant (yellow, PDB ID 7RL2) superimposed on to the s-warfarin complex (green, PDB ID 1OG5) of CYP2C9. The s-warfarin is shown in gray sticks, whereas losartan in the active site of *8 complex is shown as cyan stick representation. The sidechain of F476 is shown located near s-warfarin in yellow (*8) and green (s-warfarin bound) sticks. Heme is shown in red sticks.

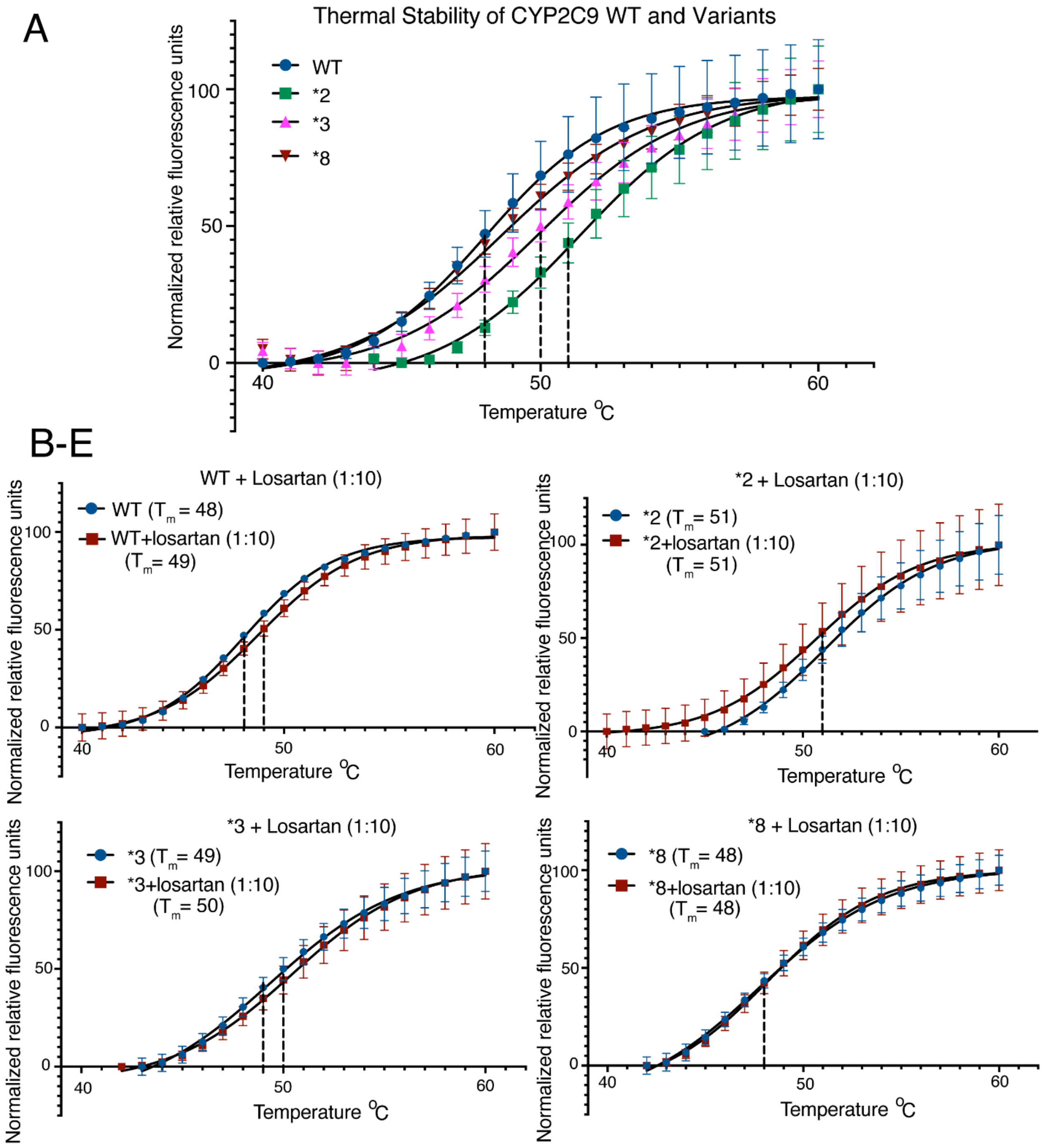

<u>Figure S3</u>: Thermal stability assay of CYP2C9 WT and the variants. A) The unfolding curve of apo CYP2C9 WT (blue) with T_m = 48 °C, *2 variant (green) with T_m = 51 °C, *3 variant (pink) with T_m = 50 °C and *8 variant (red) with T_m = 48 °C. (B-E) The unfolding curves for WT and variants in the presence and absence of losartan in a 1:10 molar ratio (protein: losartan). The melting temperature is highlighted with broken line.

**Protein Name**	**Apo Protein *Tm* (˚C)**	**Protein + Losartan (1:10 molar ratio) *Tm* (˚C)**	**Δ *Tm***
CYP2C9 WT	48.02 ± 0.20	48.71 ± 0.19	+0.69
CYP2C9 *2	51.14 ± 0.27	50.67 ± 0.35	-0.47
CYP2C9 *3	49.33 ± 0.68	50.40 ± 0.38	+1.07
CYP2C9 *8	47.81 ± 0.48	48.20 ± 0.19	+0.39

Table S1: Melting temperatures of CYP2C9 WT, *2, *3 and *8 in the absence (apo) or presence of the drug substrate losartan in a 1:10 molar ratio. The difference in temperature is represented by ΔT_m.

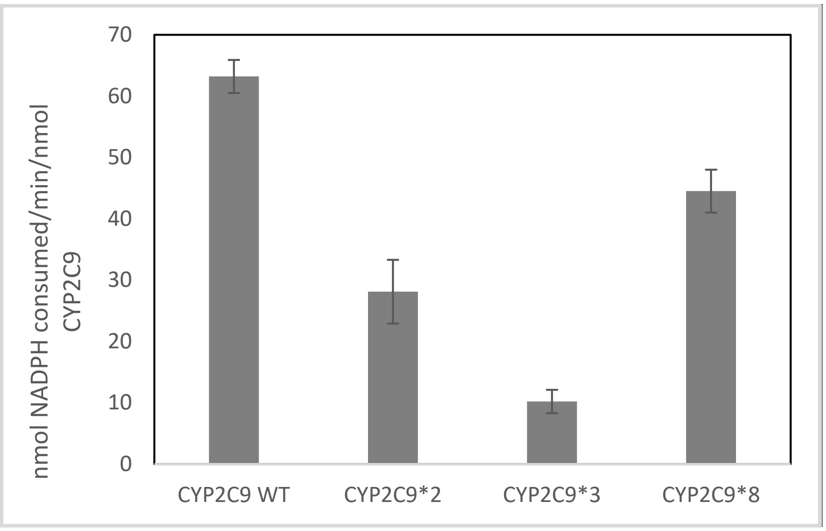

Figure S4: The amount of NADPH consumed in nmol per minute per nmol CYP2C9 enzymes in the presence of 10 µM losartan.

<u>Table S2</u>: Crystallographic data collection and refinement statistics.

Protein	CYP2C9*8
Ligand	Losartan
PDB	7RL2
Crystal Space group	*I222*
**Crystal Unit Cell Parameters**	
a (Å)	75
b (Å)	143.2
c (Å)	163.8
$\alpha = \gamma = \beta$ (°)	90
**Data Collection Statistics.**	Values for highest resolution shell are shown in parentheses.
Light source and beamline	SSRL 12-2
Wavelength (Å)	0.9
Resolution range (Å)	40.2-2.23 (2.35-2.23)
Completeness (%)	98.6 (99.5)
Redundancy	7.4 (8.2)
Mean I/sigma (I)	8.6 (1.7)
R_{merge}	0.128 (1.7)
Total reflections	42981

**Refinement Statistics.**	
R-factor (%)	17 (42)
R_{free} (%)	22 (44)
**RMS Deviations**	
Bond lengths (Å)	0.01
Bond angles (°)	2.2
Average B factor (Å^2)	59.6
**Ramachandran Plot**	
Favored (%)	95.3
Outliers (%)	0
**Number of Atoms**	Average *B*-factors (Å^2) are in parentheses.
Protein	3625 (62.83)
Heme	43 (40.1)
Solvent	125 (62.19)
Losartan	60 (59.5)
